# A Differential Profile of Biomarkers between Patients with Atrial Fibrillation and Healthy Controls

**DOI:** 10.3390/jpm12091406

**Published:** 2022-08-30

**Authors:** Ana Merino-Merino, Ruth Saez-Maleta, Ricardo Salgado-Aranda, Daniel AlKassam-Martinez, Virginia Pascual-Tejerina, Javier Martin-Gonzalez, Javier Garcia-Fernandez, Jose-Angel Perez-Rivera

**Affiliations:** 1 Cardiology Department, Hospital Universitario de Burgos, 09006 Burgos, Spain; 2 Clinical Analyses Department, Hospital Universitario de Burgos, 09006 Burgos, Spain; 3 Cardiology Department, Hospital Clínico San Carlos, 28040 Madrid, Spain; 4 Clinical Analyses Department, Hospital Central de Asturias, 33011 Oviedo, Asturias, Spain; 5 Cardiology Department, Hospital Virgen de la Salud, 45004 Toledo, Spain; 6 Universidad Isabel I, 09003 Burgos, Spain

**Keywords:** atrial fibrillation, biomarkers, NT-proBNP

## Abstract

Atrial fibrillation (AF) is explained by anatomical and electrophysiological changes in the atria determined by high pressure, dilatation, infiltration and inflammation in the myocardium. There are some biomarkers implicated in these processes, namely, NT-proBNP, high sensitivity troponin (Hs-Tn), urate, galectin-3, ST2, C reactive protein and fibrinogen. The aim of this study was to assess differences in these biomarkers between patients with AF and healthy controls. We designed a cross-sectional study consecutively including all patients undergoing electrical cardioversion in our hospital for persistent AF and matched healthy controls. We included 115 patients with persistent non-valvular AF and 33 healthy subjects. The biomarkers NT-proBNP, ST2 and Hs-Tn T were significantly related to the presence of AF (1054 ± 833.30 vs. 58.31 ± 59.40, *p* < 0.001; 35.43 ± 15.89 vs. 27.43 ± 10.95, *p* < 0.001 and 10.25 ± 6.11 vs. 8.42 ± 6.85, *p* < 0.001, respectively). NT-proBNP was the best biomarker differentiating AF patients (area under the curve 0.995). The best NT-proBNP cut-off point to differentiate AF was 102 pg/mL; for Hs-Tn T it was 11.5 ng/L and for ST2 it was 37.7 ng/mL. It is possible that these biomarkers intervene at the onset of AF and have no role in AF maintenance.

## 1. Introduction

Atrial fibrillation (AF) is the most frequent arrhythmia. It is explained by anatomical and electrophysiological changes in the atrial myocardium determined by high pressure, dilatation, infiltration and inflammation. Identification of biomarkers associated with AF may advance knowledge of AF, increasing our understanding of the pathophysiological mechanisms of the arrhythmia. Including biomarkers associated with AF in risk scales may yield predictions of AF risk with more precision in the future. Moreover, biomarkers may be used in the development of pharmacological pathways for AF preventive therapies [1].

Some biomarkers are implicated in processes involved in AF onset and progression, namely, NT-proBNP (implicated in mechanical stress and myocardial stretch), high sensitivity troponin T (Hs-Tn T) (a myocardial damage biomarker), urate (associated with oxidative stress), galectin-3 and ST2 (implicated in remodeling and fibrosis), C reactive protein (CRP) and fibrinogen (implicated in inflammation) [2,3,4].

The relationship between biomarkers and AF has been previously shown [5], but the potential value of combining several biomarkers to achieve an integrated assessment is still not fully established [6].

The aim of this study was to assess differences in these biomarkers between AF patients and healthy subjects.

## 2. Materials and Methods

### 2.1. Design and Population

This cross-sectional study included all consecutive stable patients presenting with non-valvular persistent AF, non-urgently submitted to our unit for electrical cardioversion between 17 April 2015 and 14 July 2017. We selected 1:3 aged-paired controls.

An echocardiogram performed at our Cardiac Image Unit in the 6 months prior to the electrical cardioversion was required (main echocardiographic measurements are shown in Supplementary Table S1). We excluded patients with significant structural cardiac abnormalities (moderate or severe valvular disease, valvular prosthesis, history of LVEF less than 40%, hypertrophic cardiomyopathy and infiltrative cardiomyopathy), presence of atrial flutter or arrhythmias other than AF, previous cardioversion or pulmonary vein ablation, patients with clinical instability and asymptomatic patients. None of the controls had a history of AF or any other cardiovascular disease in any of its forms.

In the basal clinical interview, a cardiologist checked the inclusion and exclusion criteria and recruited patients and controls agreeing to sign the informed consent form. The local Ethics Committee’s approval was obtained for this study (reference number, CEIC-1407). The study was performed in accordance with the Declaration of Helsinki. Prior to cardioversion, blood samples of all patients were obtained. Blood samples of the controls were obtained on the day of inclusion. The biomarkers determined by our center’s laboratory were NT-proBNP, Hs-Tn T, galectin-3, ST2, fibrinogen, urate and CRP. Glomerular filtration rates were estimated using the CKD-EPI equation.

### 2.2. Statistical Analysis

SPSS version 20.0 for Windows (IBM, Chicago, IL, USA) was used to perform the statistical analysis. Quantitative variables were expressed as mean and standard deviation or medians, and as interquartile ranges when a normal distribution was not observed, as per the Kolmogorov–Smirnov goodness-of-fit test. Qualitative variables were expressed as frequency and percentage. In order to assess differences in biomarker levels and clinical variables in subjects with or without AF, a univariate analysis was performed using the *t*-test for normal quantitative variables, the U–Mann–Witney test for non-normal quantitative variables, and the Chi-square test for qualitative variables. Receiver operating characteristic (ROC) curves were obtained to assess the biomarkers’ most accurate diagnostic cut-off values. The best cut-off point corresponded to the maximum vertical distance between the ROC curve and the diagonal line. Finally, we performed multivariate analysis using logistic regression analysis including those variables that showed statistical significance (*p* < 0.05) in the univariate analysis.

## 3. Results

### 3.1. Clinical Differences between Cases and Controls

We included 115 patients with AF and 33 healthy controls. Differences in clinical characteristics between cases and controls are shown in Table 1. Only the male sex was significantly related to the presence of AF (71.30% vs. 51.51%; *p* = 0.033).

### 3.2. Analytical Differences between Cases and Controls

Differences in analytical characteristics (including biomarkers) between cases and controls observed in the univariate analysis are shown in Table 2. Renal function, measured as creatinine levels and glomerular filtration (0.95 ± 0.19 mg/dl vs. 0.79 ± 0.12 mg/dl; *p* < 0.001 and 79.45 ± 15.23 mL/min vs. 90.54 ± 10.07 mL/min; *p* < 0.001, respectively), was significantly related to the presence of AF. Biomarkers NT-proBNP (1054.20 ± 833.30 pg/mL vs. 58.31 ± 59.40 pg/mL; *p* < 0.001), ST2 (35.43 ± 15.89 ng/mL vs. 27.43 ± 10.95 ng/mL; *p* < 0.001) and Hs-Tn T (10.25 ± 6.11 ng/L vs. 8.42 ± 6.85 ng/L; *p* < 0.001) were also significantly related to the presence of AF.

### 3.3. ROC Test

**Figure 1:**
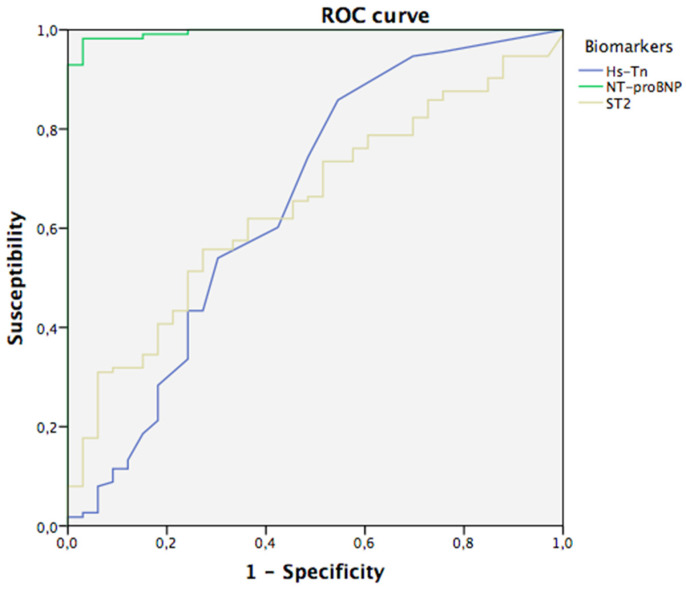
ROC curves. Hs-Tn: High sensitivity troponin.

To assess the biomarkers’ yield, we performed ROC tests including those biomarkers that showed a significant relationship with the presence of AF in the univariate analysis. The area under the ROC curve for NT-proBNP was 0.995, for Hs-Tn T it was 0.655 and for ST2 it was 0.648. ROC curves are shown in Figure 1.

We used the deLong test to compare the AUCs of three biomarkers. NT-proBNP was significantly better than Hs-Tn T (*p* < 0.001) and better than ST2 (*p* < 0.001), but there were no significant differences between Hs-Tn T and ST2 (*p* = 0.99). NT-proBNP was the best biomarker for differentiating patients with AF.

From this test, we obtained the best cut-off points to differentiate cases and controls for three biomarkers. The best cut-off point for NT-proBNP was 102 pg/mL (99% sensibility and 76% specificity), for Hs-Tn T it was 11.5 ng/L (28% sensibility and 82% specificity) and for ST2 it was 37.7 ng/mL (40% sensitivity and 82% specificity).

### 3.4. Multivariate Analysis

To perform multivariate analysis we included those clinical variables and biomarkers that showed a statistical significance in the univariate analysis: male sex, glomerular filtration (we included this instead of creatinine because it provides information regarding renal function that is more accurate) and biomarkers Hs-Tn T, NT-proBNP and ST2. We present the results with no dichotomized biomarkers levels, and then with dichotomized biomarkers levels, using the best cut-off points from the ROC curve.

In the first analysis (Table 3), NT-proBNP was the only variable independently related to the presence of AF (odds ratio 1.03; 95% confidence interval 1.01–1.04; *p* < 0.001). 

In the second analysis, NT-proBNP was the only variable independently related to the presence of AF (odds ratio 442.16; 95% confidence interval 46.27–4224.83; *p* < 0.001). The odds ratio in this case was remarkably high. We found two possibilities that explain this, including the small sample size and that the vast majority of patients (97.39%) showed NT-proBNP levels above the cut-off point. As a result, in samples such as ours, the odds ratio is particularly high and should not be taken into account.

## 4. Discussion

In our sample, we detected higher levels of cardiac biomarkers in AF patients than in healthy controls. NT-proBNP showed the best performance in discriminating cases and controls.

We found a higher proportion of men in the patient group with AF than in healthy controls (71.30% vs. 51.51%; *p* = 0.033). According to previous studies, this might be due to the high prevalence of cardiovascular risk factors among men [7]. We paired controls by age, but not by sex; therefore, differences may have been found by chance. Nevertheless, we included sex in the multivariate analysis to avoid confounding factors.

NT-proBNP, ST2 and Hs-Tn T were the only biomarkers significantly related to the presence of AF. Similar to our study, NT-proBNP has previously been independently related to the presence of AF [8,9]. Moreover, it has been demonstrated that the development and progression of AF (from paroxysmal to persistent) is associated with a gradual increase in NT-proBNP levels [10].

Although NT-proBNP levels are related to a higher risk of AF, cut-off points and treatments based on those points are not yet established. In our study, the best NT-proBNP cut-off point was 102 pg/mL (99% sensitivity and 76% specificity). Palà et al. [11] noted a similar cut-off point for NT-proBNP (95 pg/mL, 95% sensibility and 66.2% specificity). The association between NT-proBNP and the presence of AF can be explained by atria remodeling (in which NT-proBNP is implicated) when it is expressed secondary to atrial distension and dilatation. Our research group has previously shown a relationship between NT-proBNP and recurrences of AF. Patients with persistent high values of this biomarker have active processes of atrial stretch, remodeling and fibrosis; these mechanisms are probably the most important contributors to AF maintenance. Furthermore, it seems that a new onset of AF can reactivate inflammation and fibrosis in the acute phase, and over the time this mechanism might decrease [12].

ST2 has previously been related to AF and has been shown to have higher values in patients with persistent AF compared to patients with paroxysmal AF, which can translate progression of the AF [13]. In contrast, ST2 values were significantly higher in patients with persistent and permanent AF compared to patients in sinus rhythm; however, no significant differences were found between persistent and permanent AF [14].

In our study, the best ST2 cut-off point to discriminate AF was 37.7 ng/mL (40% sensitivity and 82% specificity). The association between ST2 and AF can be explained by the implication of ST2 in fibrosis and remodeling processes that initiate and maintain AF. A performance algorithm has been described in maintaining sinus rhythm based on ST2 values [15]. This algorithm was based on the hypothesis that elevated ST2 levels translate into excess myocardial fibrosis. Therefore, patients with high ST2 levels (considering the cut-off point of 35 ng/mL) would not benefit from performing electrical cardioversion and should be evaluated in a specialized consultation to assess pulmonary vein ablation [16].

Hs-Tn T has not been classically related to AF, but it has been shown that high levels of this biomarker are associated with the incidence of AF [17]. Increased levels of Hs-Tn T in AF patients in our study was probably a result of the myocyte damage that can occur in AF.

In our study, the best Hs-Tn T cut-off point to discriminate AF was 11.5 n/L (28% sensitivity and 82% specificity). A relationship between Hs-Tn and AF has been previously shown. A metanalysis including 27 studies showed significantly higher Hs-Tn levels in AF patients than in subjects without AF [18].

In a recent study that included more than 3000 patients with mild or moderate chronic kidney disease with a 7-year follow-up, Hs-Tn T values were associated with a higher risk of AF onset [19]. Another study with 241 AF patients and 824 subjects with no cardiovascular disease showed increased Hs-Tn T levels in those with AF. Moreover, patients with persistent AF showed higher levels of Hs-Tn T than those with paroxysmal AF. That study also showed a relationship between Hs-Tn T and the presence of low voltage areas in the left atria. The authors explained that these areas translate remodeling and fibrosis zones with proapoptotic processes; however, the progression of AF does not necessarily translate the destruction of cardiomyocytes [20].

In a multicenter study of hospitalized patients with COVID-19, troponin and NT-proBNP levels were significantly higher in patients with a history of AF than in patients without a history of AF. Nevertheless, there were no differences in other biomarkers, such as CRP [21].

Several biomarkers (urate, galectin-3, fibrinogen and CRP) did not show any relationship with the presence of AF in our sample. Our AF patients presented different AF durations, but they all had persistent AF. It is possible that these biomarkers intervene at the onset of AF and have no role in AF maintenance; it can also be explained by the small sample size.

Regarding the rest of the analytical values, creatinine levels were higher in AF cases than in controls and glomerular filtration were lower in AF cases than in controls. A bidirectional relationship exists between kidney disease and cardiovascular disease (including AF) [22]. In fact, chronic kidney disease is a predictor of cardiovascular disease as well as the onset of AF; it is two or three times more likely than in patients without chronic kidney disease [23]. On the other hand, the presence of AF is related to the progression of kidney disease [24].

Our study has some limitations. We only included patients with symptomatic persistent AF; therefore, our results cannot be extrapolated to other populations. We did not take differences in AF duration between our patients into account. We did not analyze different variables, such as previous electrical cardioversion or pulmonary veins ablation, which could impact biomarker levels and might also be affected by the presence of AF. Finally, given our study design, we cannot elucidate whether the increase in biomarker levels in our study’s sample population was a cause or a consequence of AF.

On the other hand, to our knowledge this is the first study analyzing a wide battery of biomarkers in AF patients and healthy controls. The identification of pathophysiological phenomena of atrial remodeling could be useful in detecting individuals at risk of developing AF. Using biomarkers to detect AF risk could facilitate the application of more exhaustive diagnostic procedures for affected patients.

## 5. Conclusions

In our sample, NT-proBNP, ST2 and Hs-Tn T were related to the presence of AF. NT-proBNP showed the highest yield in differentiating patients with AF from healthy subjects, and was the only biomarker independently related to the presence of AF.

## Figures and Tables

**Table 1:** Clinical differences between cases and controls.

	Cases (N = 115)	Controls (N = 33)	*p*
Age	63 ± 9	62 ± 10	0.464
Men	82 (71.30%)	17 (51.51%)	0.033
Hypertension	65 (56.52%)	15 (45.45%)	0.261
Diabetes	15 (13.04%)	4 (12.12%)	0.889
COPD	5 (4.34%)	1 (3.03%)	0.729
Smoking	19 (16.52%)	8 (24.24%)	0.322
CRD	2 (1.739%)	1 (3.03%)	0.643
Stroke	3 (2.60%)	1 (3.03%)	0.895
Previous myocardial infarction	6 (5.21%)	1 (3.03%)	0.602
OSAHS	10 (8.69%)	2 (6.06%)	0.625

CRD: Chronic Renal Dysfunction; COPD: Chronic Obstructive Pulmonary Disease.

**Table 2:** Analytical differences between cases and controls.

	Cases (N = 115)	Controls (N = 33)	*p*
NT-proBNP (pg/mL)	1054.20 ± 833.30	58.31 ± 59.40	<0.001
Galectin-3 (ng/mL)	16.87 ± 4.89	22.71 ± 21.94	0.139
ST2 (ng/mL)	35.43 ± 15.89	27.43 ± 10.95	<0.001
Fibrinogen (mg/dl)	329.40 ± 75.87	315.33 ± 73.31	0.346
Hs-Tn T (ng/L)	10.25 ± 6.11	8.42 ± 6.85	<0.001
Urate (mg/dl)	6.11 ± 1.45	6.38 ± 7.68	0.845
CRP (mg/L)	5.06 ± 14.8	2.46 ± 2.10	0.326
Hemoglobin (g/dl)	14.97 ± 1.43	14.56 ± 1.29	0.150
Creatinine (mg/dl)	0.95 ± 0.19	0.79 ± 0.12	<0.001
Glomerular filtration (mL/min)	79.45 ± 15.23	90.54 ± 10.07	<0.001

CRP: C-reactive protein; Hs-Tn T: High sensitivity troponin T.

**Table 3:** Multivariate analysis.

	Odds Ratio	95% Confidence Interval	*p*
NT-proBNP (pg/mL)	1.03	1.01–1.04	<0.001
ST2 (ng/mL)	1.25	0.88–1.79	0.215
Hs-Tn T (ng/L)	0.81	0.61–1.08	0.159
Men	37.60	0.51–2770.86	0.098
Glomerular filtration (mL/min)	0.93	0.82–1.04	0.220

Hs-Tn T: High sensitivity troponin T.

## Data Availability

Raw data were generated at the University Hospital of Burgos. Derived data supporting the findings of this study are available from the corresponding author, J.-A.P.-R., on request.

## Supplementary Materials

The following supporting information can be downloaded at: https://www.mdpi.com/article/10.3390/jpm12091406/s1, Table S1: Echocardiographic measurements in patients with atrial fibrillation.
