# Supplementary material for: A Differential Profile of Biomarkers between Patients with Atrial Fibrillation and Healthy Controls
Source: J Pers Med. 2022 Aug 30;12(9):1406. doi: 10.3390/jpm12091406 (PMC9503201; doi:10.3390/jpm12091406)
Supplement: Supplementary file 1 [file jpm-12-01406-s001.zip › jpm-1875202-supplementary.pdf]

Supplementary Table S1. Echocardiographic measurements in patients with atrial fibrillation.

Indexed left atrium area (mm/m^2)	25,15 ± 5,76
Indexed left atrium volume (ml/m^2)	75,62 ± 28,73
Interventicular septum (mm)	11,11 ± 2,31
E/é lateral	15,24 ± 44,78
Indexed left ventricular telediastolic volume (ml/m^2)	51,89 ± 10,81
Indexed left ventricular telesystolic volume (ml/m^2)	35,38 ± 10,46
Left ventricular ejection fraction using the biplane Simpson method (%)	59,41 ± 8,40
